# Repositioning Tradition: A Narrative Review and Expert Opinion on the Clinical Positioning of Glimepiride and Metformin in Nepal's Type 2 Diabetes Mellitus (T2DM) Landscape

**DOI:** 10.7759/cureus.110712

**Published:** 2026-06-12

**Authors:** Sabin Chaulagain, Madhu Pandey, Subesh Saurav, Dipak Malla, Krishna Kumar Agrawal, Saurav Khatiwada, Taranath Koirala, Aushili M

**Affiliations:** 1 Internal Medicine/Endocrinology, Scheer Memorial Adventist Hospital, Banepa, NPL; 2 Endocrinology, Norvic International Hospital, Kathmandu, NPL; 3 Endocrinology, Manmohan Hospital, Birtamod, NPL; 4 Endocrinology, National Academy of Medical Sciences, Kathmandu, NPL; 5 Nephrology, Universal College of Medical Sciences, Bhairahawa, NPL; 6 Internal Medicine/Endocrinology, Chitwan Medical College, Bharatpur, NPL; 7 Endocrinology, Pokhara Super Speciality Health Clinic, Pokhara, NPL; 8 Scientific Services, USV Private Limited, Mumbai, IND

**Keywords:** cost-effective, glycemic control, hypoglycemia, recommendations, tolerability

## Abstract

The prevalence of type 2 diabetes mellitus (T2DM) is rising in Nepal, making the glimepiride-metformin fixed-dose combination (FDC) a commonly used treatment due to its complementary mechanisms of action, effectiveness, and cost-efficiency. This expert opinion aims to identify diverse patient populations most likely to benefit from this combination and guide evidence-based, individualized diabetes management in Nepal. An expert advisory panel consisting of nine clinicians from Nepal convened across two meetings (held in February and March 2025) to share their perspectives on the glimepiride and metformin FDC. Their insights were based on real-world practice patterns, treatment challenges, and local prescribing dynamics to develop clinically relevant recommendations. The expert panel discussions, supported by clinical data, demonstrated that the glimepiride-metformin FDC is a widely used, effective, and affordable option for managing T2DM. It is particularly beneficial for patients requiring rapid glycemic control, those recently diagnosed, and individuals in resource-limited settings. The FDC was found to provide significant glycated hemoglobin (HbA1c) reductions, tolerability at low doses, and improved treatment adherence due to its simplified regimen. While challenges such as the risk of hypoglycemia, weight gain, and limited formulation availability persist, the combination remains a preferred choice in settings where cost and availability are critical concerns. The panel emphasized the importance of individualized dose titration and regular follow-up to optimize outcomes. The glimepiride and metformin FDC continue to be a clinically effective, affordable, and widely applicable treatment option for T2DM in Nepal, particularly suited to meet the needs of diverse patient populations, specifically in resource-limited settings.

## Introduction and background

Diabetes mellitus (DM) has become a global health epidemic in the last few decades. As per the International Diabetes Federation (IDF), 589 million adults had diabetes in 2024, expected to increase to 853 million by 2050, with the disease responsible for 3.4 million deaths in 2024, one every nine seconds [1]. Type 2 DM (T2DM), a chronic metabolic condition, is increasingly emerging as a potential epidemic worldwide. This escalating disease burden can add to the accelerated rate of complications, decreasing quality of life, and early mortality [2].

In Nepal, the trend of T2DM prevalence has been a cause for concern, as it increased from an estimated 7.75% in 2010-2015 to 11.24% in 2015-2020, indicating the need for immediate and affordable management options [3].

The cornerstone of T2DM management generally comprises lifestyle changes in combination with pharmacotherapy for optimal glycemic control and the prevention or delay of long-term macrovascular and microvascular complications [4]. Metformin, a biguanide, is the first-line treatment of choice for T2DM in adults. It decreases both basal and postprandial glucose (PPG) concentrations by limiting hepatic glucose output, reducing intestinal absorption, and enhancing insulin sensitivity. It is used extensively as monotherapy or with other drugs when lifestyle changes alone are not adequate to provide glycemic control [5].

In recent years, newer classes of glucose-lowering agents, including sodium-glucose cotransporter-2 inhibitors (SGLT2i) and glucagon-like peptide-1 receptor agonists (GLP-1 RA), have gained prominence due to their demonstrated cardiovascular (CV) and renal benefits in specific patient populations [6,7]. Consequently, many international guidelines recommend these agents, particularly in individuals with established CV disease, chronic kidney disease (CKD), or high CV risk [7,8].

Modern sulfonylureas (SUs), such as glimepiride, remain widely used due to their glucose-lowering efficacy. While some studies have explored potential pleiotropic effects (anti-inflammatory or neuroprotective properties), these findings are not yet supported by consistent or conclusive evidence. Current guidelines generally position SUs as an add-on to metformin in current diabetes management [9-11].

Among these, the glimepiride (a second-generation SU) and metformin combination is most prescribed for better blood glucose control because it can counteract "insulin secretion disorder" and "insulin resistance," respectively [12]. Fixed-dose combinations (FDCs) of these agents are widely available in Nepal and are commonly prescribed in both primary and specialist care settings [13]. The combination has shown high reductions in glycated hemoglobin (HbA1c) values, fasting plasma glucose (FPG), and PPG, and has emerged as a cost-effective, widely used alternative with preference in resource-limited settings [14]. While much of the available evidence is derived from global studies, its applicability to the Nepal population should be interpreted with caution, highlighting the need for more country-specific data to inform clinical decision-making.

In line with this, to better understand local practices and patient needs, this expert opinion document synthesizes the collective insights from leading clinical experts in Nepal regarding the clinical use and positioning of the glimepiride-metformin FDC. The primary objective of this expert advisory is to identify and profile the types of patients who are most likely to benefit from this combination, thereby facilitating optimized treatment outcomes and contributing to evidence-based prescribing patterns in Nepal.

## Review

Methods

An advisory group of nine clinical experts participated in two advisory board meetings held in Nepal between February and March 2025. The study was designed as a qualitative expert consensus to evaluate the clinical positioning of the glimepiride-metformin FDC in diabetes management in Nepal.

Prior to the meetings, a targeted literature search was conducted to support the development of key discussion themes. Relevant evidence was identified through searches of PubMed/MEDLINE, Embase, and Google Scholar, supplemented by relevant clinical practice guidelines and grey literature pertaining to diabetes management in Nepal and South Asia. The search used combinations of keywords, including type 2 diabetes, glimepiride, metformin, fixed-dose combination, and dual therapy. The evidence collection was exploratory and used to inform discussion points. Additional relevant publications were identified through manual screening of reference lists.

Further experts were selected based on predefined criteria, including specialization in diabetology/endocrinology, with a minimum of 10 years of experience, and representation from diverse clinical practice settings across Nepal.

The 10 key clinical issues were developed through literature review, guidelines, and through experts' discussions, which were focused on the perception and positioning of the glimepiride-metformin FDC in diabetes management in Nepal. The panel also shared their expertise on the real-world prescribing patterns, challenges in tailoring treatment, and the competitive pharmaceutical landscape. In-depth discussions and expert analyses were used to formulate informed opinions.

Results

Considering the advantages of improved compliance and cost-effectiveness, the use of FDC therapy of modern SU and metformin combination has increased significantly in recent years. These FDC formulations enhance patient adherence by reducing pill burden compared to separate agents and offer the potential to achieve glycemic targets more effectively with lower individual drug doses. This strategy not only enhances the convenience of treatment but also assists in alleviating the adverse effects of high-dose monotherapy while preserving strong blood glucose control [15,16]. Several consensus and guidelines recommend adding SUs to metformin in settings with high diabetes prevalence and limited healthcare resources [2,17-19].

FDC of Glimepiride and Metformin

Statement: The FDC of glimepiride and metformin is routinely prescribed for the management of T2DM in patients requiring rapid glycemic control and is a cost-effective option.

Summary of discussion: Metformin remains the effective therapy for T2DM, with glimepiride frequently added when glycemic targets are not achieved. Glimepiride is used as a second-line agent in selected patients with T2DM who retain adequate β-cell function, as its efficacy depends on endogenous insulin secretion. In resource-limited settings, such as rural India and Nepal, the affordability of glimepiride-metformin FDCs makes them a practical and widely used choice. Common dosing ranges from 4-6 mg/day, though lower doses (e.g., ≤ 2 mg/day) are often preferred to minimize the risk of hypoglycemia. When dosed appropriately, the combination is considered safe and effective. The FDC also simplifies treatment regimens, which is particularly beneficial for patients with poor treatment literacy or adherence challenges. In Nepal, government hospital physicians often prescribe glimepiride-metformin FDCs due to socioeconomic constraints, patient frailty, and limited access to newer oral antidiabetic drugs (OADs).

Clinical evidence: A randomized, open-label, multicenter study in Korea compared glimepiride-metformin FDC therapy with up-titrated metformin monotherapy in patients with T2DM. The FDC group demonstrated significantly better glycemic control, with a larger mean reduction in HbA1c (-1.2% vs. -0.8%; p < 0.0001) and FPG (-35.7 vs. -18.6 mg/dL; p < 0.0001). A greater percentage of patients in the FDC group achieved target HbA1c levels by the study's end [16]. In the retrospective multi-center cross-sectional study (GLIMPSE study), newly diagnosed T2DM patients treated with metformin-glimepiride FDC for three months reported significant improvements in HbA1c (from 8.3% to 7.3%), fasting blood glucose (FBG) (from 172.8 to 134.2 mg/dL), and PPG (from 245.9 to 187.8 mg/dL), with most patients rated as having excellent or good treatment outcomes [20]. A multicenter study in Nepal evaluated adults with T2DM and found that a combination therapy of glimepiride (1 mg) and metformin (1,000 mg sustained release) significantly improved glycemic control. Key outcomes included notable reductions in HbA1c (-2.45%), FPG (-64.53 mg/dL), and PPG (-102.64 mg/dL), all statistically significant. Physicians rated the treatment highly, with over 94% showing good-to-excellent efficacy and over 96% good tolerability [21].

Additionally, an observational study confirmed significant improvements in glycemic parameters with glimepiride-metformin FDC therapy (p < 0.001) [22]. A pharmacoeconomic analysis indicated that this combination is more cost-effective for lowering HbA1c and FPG in T2DM patients [23]. According to the pooled cohort analysis, long-term use of metformin and SUs, individually or in combination, was associated with a reduced risk of microvascular complications in individuals with newly diagnosed patients with T2DM, sustained for up to approximately one decade [24]. Real-world data from the Joint Asia Diabetes Evaluation (JADE) register indicate that a majority of patients with T2D are managed with oral glucose-lowering drugs, with approximately 60% receiving SUs, commonly in combination with metformin. The favourable glycaemic control, safety profile, and affordability of SUs, including gliclazide, reinforce their continued role as a key therapeutic option in the management of T2D in the region [25].

In elderly patients, the choice of an SU should be individualized, taking into account glycaemic requirements, safety, and ease of use [26,27]. Modified-release gliclazide (gliclazide MR) has been associated with a lower risk of hypoglycaemia in this age group [28]. It is also worth noting that a FDC of gliclazide MR and metformin is currently unavailable, which may affect treatment convenience and adherence in clinical practice.

A metabolic fulcrum-based approach has been proposed to guide oral hypoglycemic agents selection in low- and middle-income countries. It categorizes T2DM patients as eubolic, predominantly catabolic, or maladaptive anabolic based on phenotypic features alone, allowing rational therapy selection. This approach supports the effective use of SUs, particularly in eubolic individuals, to facilitate timely glycaemic control [29].

Overall, the combination of glimepiride and metformin is recognized as a clinically effective treatment option with cost-effectiveness [30,31].

Glimepiride-Metformin in Specific Cases

Statement: Glimepiride and metformin FDC may be considered in selected patients, particularly in lean individuals, newly diagnosed individuals with markedly elevated HbA1c/PPG requiring rapid glycemic control, and in resource-limited settings where cost restricts the use of newer agents, such as SGLT2i, DPP-4 inhibitors, or GLP-1 receptor agonists.

Summary of discussion: In lean patients with T2DM, glimepiride is commonly used as a second-line agent due to its insulin-secreting effect that complements metformin's glucose-lowering effects. The FDC of glimepiride and metformin is particularly effective in newly diagnosed patients or those with significantly elevated HbA1c or PPG levels, providing potent and immediate glycemic control. For patients challenged by multiple pills, once-daily glimepiride-metformin FDC enhances adherence compared to multiple separate doses of newer antidiabetic agents. However, in patients with CKD, caution is warranted when using glimepiride because of heightened hypoglycemia risk. Dose adjustments guided by eGFR are essential, and safer SUs such as gliclazide or glipizide may be a preferred alternative. In individuals with CV risk (e.g., ischemic heart disease), gliclazide is generally considered safer than glimepiride, although the latter can still be used with careful monitoring.

Clinical evidence: A systematic review and meta-analysis of clinical trials comparing glimepiride and DPP-4 inhibitors, both with metformin as second-line therapy for T2DM, demonstrated that glimepiride consistently achieved better glycemic control. Patients on glimepiride demonstrated a significantly greater HbA1c reduction (weighted mean difference: -0.12; 95% CI: -0.16 to -0.07), along with improved FPG and a greater proportion reaching HbA1c < 7%, highlighting its superior antihyperglycemic effect compared to DPP-4 inhibitors [30]. In a real-world study of 941 newly diagnosed T2DM patients, low-dose glimepiride (0.5 mg) with metformin 500 mg led to a 24.5% reduction in FPG, 26.5% in PPG, and 28.2% in random blood glucose over three months, with no hypoglycemia or weight gain, supporting its role in early-stage diabetes requiring rapid control [32]. A 12-week randomized multicenter trial in India also reported a greater HbA1c reduction with glimepiride (0.42%) vs. sitagliptin (0.30%) (p = 0.001), with significant improvements in FPG and PPG (p = 0.008) [33]. Further, a retrospective real-world study highlighted the widespread use and efficacy of glimepiride-metformin FDC in patients with comorbidities, indicating its suitability across disease stages [34]. Glimepiride also offers glycemic efficacy comparable to newer medications such as DPP-4 inhibitors and GLP-1 receptor agonists, but at significantly lower cost [35].

Any potential CV benefit attributed to glimepiride should be interpreted with caution, as it may primarily reflect improved glycemic control rather than a direct cardioprotective effect. Although inhibition of soluble epoxide hydrolase, which increases levels of epoxyeicosatrienoic acids, has been proposed as a possible mechanism with cardioprotective implications, this pathway remains largely theoretical in the clinical setting. Additionally, findings from major trials, such as CAROLINA and GRADE, have shown that glimepiride is non-inferior to DPP-4 inhibitors and insulin glargine in terms of major adverse CV events (MACE) and heart failure (HF) hospitalizations, supporting its CV safety even in high-risk patients [36-38]. Emerging evidence supports the CV safety of glimepiride as a second-line agent in T2DM. A nationwide population-based cohort study from Scotland reported that SU were not associated with an increased risk of MACE or all-cause mortality when compared to (DPP-4) inhibitors or thiazolidinediones (TZDs). The hazard ratios (HRs) for MACE were 1.00 (95% CI: 0.91-1.09) using Cox regression, and 1.02 (0.91-1.13) and 1.03 (0.91-1.16) using two instrumental variables. Similarly, for all-cause mortality, HRs were 1.03 (0.94-1.13), 1.04 (0.93-1.17), and 1.03 (0.90-1.17), respectively. These results suggest that SUs such as glimepiride do not carry an increased risk of major adverse CV events or all-cause mortality compared to DPP-4 inhibitors and TZDs [39]. Another, prospective cohort study involving patients with T2DM and chronic (HF demonstrated that long-term continuous use of glimepiride was associated with significantly lower CV mortality (adjusted HR: 0.34; 95% CI: 0.24-0.48; p < 0.001), all-cause mortality (adjusted HR: 0.47; 95% CI: 0.35-0.63; p < 0.001), and reduced hospitalizations for HF and atherothrombotic events (p < 0.001). These benefits were more pronounced at higher doses (2-4 mg/day), suggesting a potential dose-dependent cardioprotective effect (adjusted HR for high vs. low dose: 0.55; 95% CI: 0.31-0.99; p = 0.047) [40]. Glimepiride does not increase short-term CV risk, making it a safe option for glucose control in people with type 2 diabetes [41].

Evidence from the GRADE randomized clinical trial in 5,047 patients with early T2DM and preserved kidney function reported no significant differences in kidney outcomes over five years among glimepiride, sitagliptin, liraglutide, or insulin glargine added to metformin. Glimepiride showed a comparable eGFR decline (-1.92 mL/min/1.73 m²/year; p = 0.61) and kidney disease progression rate (12.4%; p = 0.56), confirming its renal safety in early T2DM [42].

While the glimepiride-metformin FDC effectively lowers glucose and has demonstrated CV safety, newer agents also provide additional benefits as SGLT2, GLP-1 receptor agonists, and dual agonists such as tirzepatide, found to reduce HF and kidney disease risk, promote weight loss, lower CV events, and may offer further metabolic and organ protection. Treatment choice should consider these broader benefits alongside glycemic control [43].

Stepwise Dose Titration

Statement: A combination of glimepiride and metformin should be initiated at the lowest effective dose and adjusted in a stepwise manner based on glycemic response, hypoglycemia risk, renal function, and patient adherence, with regular follow-up for guiding up-titration or down-titration.

Summary of discussion: The initiation of glimepiride-metformin therapy in T2DM patients should be tailored to individual patient profiles, considering factors such as age, renal function, beta-cell reserve, HbA1c levels, and the presence of comorbidities. In newly diagnosed patients, a common starting dose is glimepiride 2 mg combined with metformin 1,000 mg. After three to four weeks, dose adjustments should be guided by glycemic response and tolerability; this may involve down-titrating glimepiride to 1 mg or switching to alternative therapies, such as gliptins or metformin monotherapy. If glycemic control remains inadequate at 1 mg of glimepiride, the dose may be increased stepwise up to 4 mg, and in a few cases, up to 6 mg. The glimepiride and metformin combination offers a substantial HbA1c reduction of approximately 1.5-2%, which is greater than the 0.7-1% typically achieved with gliptins. Therapy should always begin with the lowest effective dose and be titrated cautiously based on clinical response and patient tolerance. In obese individuals, glimepiride is generally avoided due to its potential to cause weight gain; SGLT2i are more preferred in such cases, given their weight-reducing benefits. Failure to achieve glycemic control with this combination may necessitate therapeutic escalation or switching, particularly in patients with CKD, CV disease, insulin use, or a high risk for hypoglycemia. Frequent follow-up is crucial for monitoring both glycemic response and potential hypoglycemic episodes. Titration should be responsive to HbA1c trends, like up-titration in cases with HbA1c >8.5-9%, and down-titration when control improves. Patient education on timely food intake post-dosing is essential to minimize the risk of hypoglycemia.

Clinical evidence: Several real-world and clinical studies support the use of low-dose glimepiride and metformin FDC as an effective and well-tolerated option for managing T2DM. In a survey of 112 clinicians and 1,403 patients, 71.5% were initiated on a low dose (0.5 mg/500 mg), in some cases, with 82.7% of dose adjustments requiring up-titration, highlighting the need for individualized stepwise titration. Significant reductions in HbA1c (-1.2%), FPG (-36.5 mg/dL), and PPG (-50.2 mg/dL) were observed, with low rates of hypoglycemia (7.7%) and weight gain (9.5%) [44]. A retrospective study of 7,058 patients reported that one-third required insulin dose down-titrated following FDC initiation, with a mean HbA1c drop of 1.33%, and over 96% physicians rated efficacy and tolerability [45]. In a randomized trial of 152 patients, glimepiride-metformin outperformed glibenclamide-metformin in achieving HbA1c < 7% (44.6% vs. 26.8%; p < 0.05), and had fewer hypoglycemic events (17.1% vs. 28.9%; p = 0.047). Stepwise titration up to four tablets was effective [46].

Additionally, a multicenter observational retrospective study with 1,136 young adults (<30 years) with T2DM in India identified the glimepiride 0.5 mg and metformin 500/1,000 mg FDC as the most prescribed strength (47%). Dose titration was done as per clinical response, and notable improvements in HbA1c, FPG, and PPG levels were seen both with and without titration. Hypoglycemia occurred in only 4.8% of patients, and with no weight gain among 95.1% of patients, which further supports the start of therapy at the minimum effective dose and titration in a stepwise fashion according to glycemic response and tolerability [47].

Individualized Low-Dose Approach

Statement: Patients who are unable to tolerate higher doses of metformin and glimepiride, a low-dose combination of 0.5 mg glimepiride with 500 mg metformin, typically taken twice daily, may offer a reasonable approach to glycemic management. In patients with CKD, metformin use should be guided by renal function, and therapy must be individualized based on this.

Summary of discussion: In clinical practice, a low-dose combination of glimepiride (0.5-1 mg) and metformin (500 mg) is often preferred for lean patients, individuals with low insulin secretion, those presenting with isolated postprandial hyperglycemia, or newly diagnosed diabetes with HbA1c between 6.5 and 7.5%. This approach also considers patient perception, especially in rural or semi-urban settings where high numerical values (e.g., 1,000 mg) may be intimidating. For patients who experience hypoglycemia with 1 mg glimepiride, particularly when metformin intolerance or gliptin inefficiency limits alternatives, a 0.5 mg glimepiride and 500 mg metformin combination taken twice daily is preferred. It is noteworthy that, even at half-strength, many oral antidiabetic agents can achieve up to 80% of the efficacy seen at full doses, justifying the use of low-dose regimens in sensitive populations. In patients with CKD, metformin initiation is generally not recommended if eGFR is < 45 mL/min/1.73 m²; however, dose adjustment may be considered for those already on treatment. Emerging evidence supports the cautious continuation of metformin down to an eGFR of 30 mL/min/1.73 m² in selected cases. In elderly patients, due to frailty, hypoglycemia risk, and variable appetite, the glimepiride-metformin combination is less favoured. In this group, preferred options include gliptins, metformin (if tolerated), and gliclazide, with repaglinide being effective for postprandial control.

Clinical evidence: A case-based questionnaire survey of 1,403 patients with T2DM across 112 healthcare professionals in India reported that a low-dose combination of glimepiride (0.5 mg) and metformin (500 mg), taken twice daily, reduced HbA1c by 1.2%, FPG by 36.5 mg/dL, and PPG by 50.2 mg/dL. Hypoglycemia occurred in 7.7% and weight gain in 9.5% of patients [44].

An observational study of 941 newly diagnosed T2DM patients treated with the same low-dose combination for three months reported significant reductions in FPG (24.5%), PPG (26.5%), and random blood glucose (28.2%) without any hypoglycemia or weight gain, supporting its efficacy and tolerability in early-stage T2DM [32].

CKD considerations: The GRACE CKD real-world study in Indian adults with T2DM and CKD showed that dual FDC glimepiride and metformin improved glycaemic control and renal parameters, with dose titration required in 31.9% of patients. Reductions were observed in HbA1c, blood glucose, serum creatinine, and urine albumin-creatinine ratio, along with improved electrolytes. Therapy was well tolerated, with few mild adverse events and no hospitalizations, and was associated with better adherence and physician-reported effectiveness [48]. According to the 2022 American Diabetes Association Standards of Medical Care in Diabetes and the Kidney Disease: Improving Global Outcomes consensus, metformin is recommended for individuals with T2DM and CKD with an eGFR of ≥30 mL/min/1.73 m². For eGFR of 30-44 mL/min/1.73 m² and selected high-risk cases with eGFR of 45-59 mL/min/1.73 m², the dose should not exceed 1,000 mg daily [49]. Dosing should be individualized based on renal function and patient frailty [50]. Thus, low-dose FDCs may offer a safe, practical option for patients unable to tolerate higher doses.

Optimized Standard-Dose Therapy

Statement: The FDC of glimepiride 2 mg and metformin 1,000 mg is recommended for patients with significantly elevated blood glucose levels who prefer to avoid insulin, seek cost-effective treatment, or benefit from once-daily dosing. Total glimepiride dosage should not exceed 4 mg/day to minimize the risk of hypoglycemia, especially in elderly patients or those with CKD or autonomic dysfunction.

Summary of discussion: When considering high doses, we typically do not exceed 4 mg due to the increased risk of hypoglycemia, especially in elderly patients, those with CKD, or individuals experiencing autonomic dysfunction. On the positive side, high doses can be effective and tend to cause fewer gastrointestinal side effects. Such doses are usually initiated when patients have high blood glucose, prefer not to start insulin, face affordability issues, or benefit from once-daily dosing. It is also important to note that glimepiride should be taken before meals, as it helps control PPG levels more effectively when administered just before eating.

Clinical evidence: The GLIMPSE study, a retrospective multicenter analysis, found that the most commonly prescribed FDC for newly diagnosed T2DM patients is metformin 500 mg with glimepiride 1-2 mg. This combination targets both insulin resistance and β-cell dysfunction, offering effective glycemic control, a good safety profile, and cost-effectiveness [20]. A large real-world study of 4,858 adults with T2DM found that 10.9% were prescribed a FDC of glimepiride 2 mg and metformin 1,000 mg, with once-daily dosing used in 45.1% of cases, particularly in patients with comorbidities and complications [34]. Additionally, as per expert opinion, glimepiride 1 mg + metformin 500 mg is a preferred, effective, and well-tolerated dose for managing T2DM [31]. In a 24-week Korean trial of poorly controlled patients on low-dose metformin, the FDC reduced HbA1c (-1.2% vs. -0.8%; p < 0.0001) and greater goal attainment (HbA1c < 7% in 74.7% vs. 46.6%; p < 0.0001) than metformin up-titration. Hypoglycemia was higher (41% vs. 5.6%), but non-serious, and overall tolerability was good [22]. These glimepiride doses should be limited to ≤ 4 mg/day, especially in elderly or renally/hepatically impaired patients, due to increased hypoglycemia risk [22,51].

A systematic review indicated that both gliclazide and glimepiride effectively reduced HbA1c. Gliclazide was associated with lower overall hypoglycemia risk, but in elderly patients, it may increase the risk of severe hypoglycemia and fractures. Thus, gliclazide may be safer for middle-aged adults, while glimepiride could be more suitable for older adults when careful dose adjustment is applied [52].

Safety and Monitoring Considerations

Statement: While the glimepiride and metformin combination is a well-established and effective option for glycemic control, some considerations include the potential for hypoglycemia, particularly rebound episodes, as well as weight gain in some patients. Ensuring regular follow-up is important to optimize outcomes, and the availability of intermediate metformin strengths (such as 750 mg or 850 mg) may vary in some markets.

Summary of discussion: Hypoglycemia (including rebound episodes), weight gain, and poor long-term follow-up are key challenges with glimepiride and metformin FDC. Tablet size variability and the unavailability of intermediate metformin doses (e.g., 750 mg or 850 mg) may affect dosing flexibility and patient adherence.

Clinical evidence: In a 26-week randomized study of glimepiride vs. metformin monotherapy in pediatric T2D, both drugs reported similar HbA1c reductions (-0.54% for glimepiride vs. -0.71% for metformin). However, glimepiride led to significantly more weight gain (1.97 kg vs. 0.55 kg; p = 0.005) and a slightly higher rate of hypoglycemia (< 50 mg/dL: 4.9% vs. 4.2%). These results highlight the importance of frequent follow-up when using glimepiride to monitor and manage potential rebound hypoglycemia and weight disturbances [53]. In another four-month trial comparing glimepiride and metformin versus insulin in patients with T2D, the combination led to modest weight gain (~2.2 kg) and the lowest frequency of hypoglycemic events (2.5/patient/4 weeks) compared to metformin (3.8), glimepiride (3.3), and placebo plus insulin (5.2). This further highlights the importance of regular follow-up, particularly with SUs and insulin [54]. Access to intermediate-strength metformin formulations (e.g., 750 mg or 850 mg) is key for precise dose adjustments, especially between the common 500 mg and 1,000 mg options. Market data support that the 500 mg, 850 mg, and 1,000 mg strengths represent key segments. Initiation therapy often begins with 500 mg, while 850 mg or 1,000 mg doses are typically used for treatment escalation to improve glycemic control. These intermediate doses support flexible treatment escalation but may be limited by regional availability [55].

Long-Term Relevance of FDC

Statement: Despite the emergence of newer OADs, the glimepiride metformin combination holds a long-term role in the management of diabetes, particularly where affordability and accessibility are key considerations.

Summary of discussion: Glimepiride and metformin combination remains a reliable and frequently used option, especially in patients with high HbA1c and limited access to newer agents. While newer OADs offer added benefits, the glimepiride and metformin FDC remains valuable due to its effectiveness, affordability, and global clinical trust.

Clinical evidence: When treating T2DM, key considerations include glycemic efficacy, reduction in microvascular and macrovascular complications, safety, patient adherence, and accessibility. The ADA/EASD consensus recommends metformin as first-line therapy, with SUs as a second-line treatment, particularly when affordability is a concern. While newer agents (DPP-4 inhibitors, SGLT2i, and GLP-1 receptor agonists) offer added benefits, their high cost and limited access often pose challenges in resource-limited settings.

In contrast, newer-generation SUs, such as glimepiride and gliclazide, provide proven efficacy, safety, and cost-effectiveness, making them suitable for long-term use in low- and middle-income countries [56]. Notably, long-term use of metformin and SUs, either alone or in combination, has been associated with a significantly reduced risk of diabetic retinopathy among newly diagnosed T2DM patients, highlighting their protective and cost-effective role in early disease management. This association is generally considered to reflect improved glycemic control rather than a direct intrinsic protective effect of these drugs on the retinal microvasculature [10]. In addition, long-term use of metformin and SUs has been linked to a lower risk of microvascular complications over a decade, underscoring the importance of selecting appropriate oral therapy at the onset of diagnosis to prevent long-term complications and improve patient outcomes [24].

Overall, expert-derived key insights on the clinical use of glimepiride and metformin FDC in the management of T2DM are depicted in Table 1.

**Table 1:** Expert-derived key insights on the clinical use of glimepiride and metformin FDC in the management of T2DM CKD, chronic kidney disease; CV, cardiovascular; FDC, fixed-dose combination; GLP-1RA, glucagon-like peptide-1 receptor agonist; HbA1c, glycated hemoglobin; OADs, oral antidiabetic drugs; SGLT2i, sodium-glucose co-transporter-2 inhibitor; T2DM, type 2 diabetes mellitus This table is original and was created by the authors.

Thematic title	Point of discussion	Key insights from the experts
FDC of glimepiride and metformin	The primary reasons for prescribing glimepiride and metformin FDC over newer OADs	Glimepiride and metformin FDC provide rapid glycemic control, are affordable, and are easy to use, making them particularly suitable for resource-limited settings. It is often preferred when cost and pill burden are major considerations.
Glimepiride-metformin in specific cases	Patient scenarios/groups where glimepiride and metformin FDC are preferred over newer agents?	Preferred in newly diagnosed patients with high HbA1c or elevated postprandial glucose levels, lean individuals, and those who need immediate glycemic control. This combination is also suitable for patients with limited access to newer OADs or poor adherence.
Stepwise dose titration	Approach to dose adjustments when prescribing glimepiride and metformin FDC?	Begin therapy with the lowest effective dose of glimepiride and metformin FDC. Adjust the dosage in a stepwise manner based on glycemic response, renal function, risk of hypoglycemia, and patient tolerance. Regular follow-up is essential.
Individualized low-dose approach	Preference for low-dose glimepiride (0.5 mg/1 mg) with metformin?	Patients unable to tolerate higher doses of metformin and glimepiride, a low-dose combination of 0.5 mg glimepiride with 500 mg metformin, typically taken twice daily, may offer a reasonable approach to glycemic management. In patients with CKD, metformin use should be guided by renal function, and therapy must be individualized based on this.
Optimized standard-dose therapy	Perception of prescribing high-dose glimepiride and metformin FDC (e.g., glimepiride 2 mg/1000 mg metformin)?	The FDC of glimepiride 2 mg and metformin 1000 mg is recommended for patients with significantly elevated blood glucose levels who prefer to avoid insulin, seek cost-effective treatment, or benefit from once-daily dosing. Glimepiride doses up to 4 mg/day are generally sufficient to achieve glycemic control while minimizing the risk of hypoglycemia, especially in elderly patients or those with CKD or autonomic dysfunction.
Safety and monitoring considerations	Major challenges you face while prescribing glimepiride and metformin FDC	Key considerations include the risk of hypoglycemia, including rebound episodes, and potential weight gain in certain patients.
	Key factors influencing a shift from glimepiride and metformin to newer OADs	The shift is driven by the need to reduce the risk of hypoglycemia and weight gain, which offer added metabolic and cardio-renal benefits.
Long-term relevance of FDC	Opinion on the role of glimepiride and metformin FDC in diabetes management despite newer OADs	Despite the emergence of newer OADs, the glimepiride metformin FDC remains a valuable long-term option, especially where affordability and accessibility are priorities in the management of diabetes.

## Conclusions

The expert group, comprising leading experts from Nepal, has assessed the clinical utility, patient suitability, and market dynamics of the glimepiride and metformin FDC in the treatment of T2DM. With real-world experiences, structured discussions, and evidence-based clinical judgment, the panel declares that the glimepiride and metformin FDC remains an effective and widely utilized therapeutic option, especially in low-resource settings. Its proven efficacy in achieving rapid glycemic control, cost-effectiveness, availability in multiple strengths, in Nepal's T2DM profile; however, its use should be individualized. Current ADA guidelines favor GLP-1 receptor agonists and SGLT2i in many patients, particularly those with high HbA1c or cardiometabolic risk, and SUs should be considered within this broader, phenotype-based treatment framework.

However, the panel also notes some limitations, such as the risk of hypoglycemia, weight gain, and less favorable CV and renal outcomes compared to newer drugs. It is also important to note that any observed reductions in CV risk in studies involving sulfonylureas may be largely mediated by improved glycemic control rather than a direct cardioprotective effect, and therefore causality has not been conclusively established, particularly for agents such as glimepiride. Newer options, such as SGLT2i and GLP-1 receptor agonists, offer added benefits for certain patients. While the glimepiride-metformin combination remains widely used in Nepal, treatment should be individualized based on patient characteristics, comorbidities, and long-term safety.
